# Update on pediatric sepsis: a review

**DOI:** 10.1186/s40560-017-0240-1

**Published:** 2017-07-20

**Authors:** Tatsuya Kawasaki

**Affiliations:** Department of Pediatric Critical Care, Shizuoka Children’s Hospital, 860 Ursuhiyama, Aoi-ku, Shizuoka, Shizuoka 420-8660 Japan

**Keywords:** Sepsis, Septic shock, Pediatric, Child, Epidemiology, Surviving Sepsis Campaign, Antibiotics, Hemodynamic management, Algorithm, Prognosis

## Abstract

**Background:**

Sepsis is one of the leading causes of mortality among children worldwide. Unfortunately, however, reliable evidence was insufficient in pediatric sepsis and many aspects in clinical practice actually depend on expert consensus and some evidence in adult sepsis. More recent findings have given us deep insights into pediatric sepsis since the publication of the Surviving Sepsis Campaign guidelines 2012.

**Main text:**

New knowledge was added regarding the hemodynamic management and the timely use of antimicrobials. Quality improvement initiatives of pediatric “sepsis bundles” were reported to be successful in clinical outcomes by several centers. Moreover, a recently published global epidemiologic study (the SPROUT study) did not only reveal the demographics, therapeutic interventions, and prognostic outcomes but also elucidated the inappropriateness of the current definition of pediatric sepsis.

**Conclusions:**

With these updated knowledge, the management of pediatric sepsis would be expected to make further progress. In addition, it is meaningful that the fundamental data on which future research should be based were established through the SPROUT study.

## Background

Sepsis is a life-threatening condition which affects many children regardless of some underlying healthcare issues [1]. Sepsis is said to be one of the leading causes of death among children even in advanced countries. Although demographic data does not clearly show it, many children who are reported to die from other underlying conditions actually die directly from sepsis.

The management of pediatric sepsis was comprehensively advocated through systematic review process in the Surviving Sepsis Campaign guidelines (SSCG) 2008 [2] and 2012 [3]. Unfortunately, however, many recommendations and suggestions were still based on low quality evidence and expert consensus, and sometimes only on evidence in adult sepsis. Furthermore, the latest version of SSCG did not include a specific description of the management of pediatric sepsis [4].

This review mainly focuses on updated knowledge and hot topics regarding pediatric sepsis published after the SSCG 2012 [3].

## Definition of pediatric sepsis

For the past two decades, sepsis has been defined as “systemic inflammatory response syndrome (SIRS) caused by infection” both for adults and children [5–7]. This definition, however, has long been criticized for its too broad inclusion of milder conditions, such as influenza virus infection without any organ dysfunction. In fact, Churpek et al. revealed that nearly half of the adult patients admitted to the hospital wards fulfilled two or more SIRS criteria at least once during their ward stay [8], while Kaukonen et al. showed that about 12% of the adult ICU patients with some infection and at least one organ dysfunction were negative for the SIRS criteria but that their mortality rate was still substantial [9]. These findings imply that the SIRS criteria are not an appropriate tool to screen at-risk patients and that the severity of organ dysfunctions may be able to substitute for SIRS to identify patients with higher mortality risk.

Taking these issues into consideration, new sepsis criteria were advocated as “Sepsis-3” in 2017, which redefined sepsis as infection complicated by one or more organ dysfunctions [10]. Organ system dysfunctions are assessed with an increase in the Sequential Organ Failure Assessment (SOFA) score by 2 or more points. The main purpose of this transition is to focus on more severe patients for the recruitment in future intervention studies [10].

Unfortunately, this change in the definition of sepsis is only applied to adult population at this moment [10]. When it comes to the consensus definition in children [7], other issues have been pointed out in addition to similar criticism to the adult definition, especially regarding the pediatric SIRS and organ dysfunction criteria. The threshold of tachypnea in the pediatric SIRS criteria, for example, overlaps even the normal range for adults (e.g., 18 breaths/min for 6–12-year-old children and 14 for 13–18-year-old adolescents) [7]. The standardized criteria for each organ dysfunction [7] are not based on evidence related to clinical outcomes. Moreover, a couple of studies revealed only the moderate level of agreement, approximately two thirds, in the diagnosis of severe sepsis between physician’s clinical judgment and consensus criteria [11–13], which obviously suggests that the current consensus criteria of pediatric sepsis based on the concept of SIRS do not play an effective role in identifying clinically hazardous patients. It is strongly desirable that pediatric sepsis should be redefined on the basis of organ dysfunction scoring in accordance with the adult Sepsis-3 in near future [13, 14].

## Epidemiology

The epidemiology of pediatric sepsis varies from study to study probably because of their different era, population, and diagnostic criteria. Watson et al. first reported the population-based incidence and outcomes of severe sepsis among children under 19 years old in seven states in the USA in 1995 [1]. The incidence was 0.56 cases per 1000 children per year, which was highest among infants (5.16 per 1000) and fell dramatically with age (0.20 per 1000 among 10–14-year-olds). Their hospital mortality was 10.3%, which varied little with age and was higher among children with some co-morbidity.

More recently, a couple of studies from the USA added new findings. Following up the same population as Watson’s study [1], Hartman et al. reported that the prevalence was steadily increasing from 1995 to 2005 by 81%, which reached 0.89 cases per 1000 children in 2005 [15]. The case-fatality rate, on the other hand, dropped from 10.3% to 8.9% for that decade [15]. Based on the hospitalization database from the 44 children’s hospitals in the USA, Balamuth et al. found out that the prevalence of severe sepsis had been increasing from 3.7% to 4.4% among all the hospitalized children (18 years old or younger) between 2004 and 2012 [16]. Surprisingly, the mortality rate was significantly different between the two diagnostic populations (21.2% vs. 8.2%), one with the International Classification of Diseases, 9th edition, Clinical Modification (ICD-9) codes for severe sepsis/septic shock, the other with ICD-9 code for infection plus at least one organ dysfunction (modified Angus criteria [17]) [16]. Utilizing the same database, Ruth et al. showed the prevalence rate of severe sepsis as 7.7% (6.2% in 2004 to 7.7% in 2012) in the PICU settings of those hospitals with an associated mortality rate of 14.4% (18.9% in 2004 to 12.0% in 2012; birth to 19 years of age) [18].

A large-scale epidemiologic data recently came out from the Australia and New Zealand Pediatric Intensive Care Registry, composed of 9 PICUs and 22 general ICUs. Schlapbach et al., retrospectively investigating the registry (<16 years old) between 2002 and 2013, demonstrated that patients with invasive infection, sepsis, and septic shock accounted for 6.9%, 2.9%, and 2.1%, respectively, of the total ICU admissions. The ICU mortality rate was 3.9%, 5.6%, and 17.0% in each diagnostic group, which were much higher than 3.0% of overall ICU mortality in children. In addition, comparing the latter half of the study period with the former half, risk-adjusted mortality significantly decreased for invasive infection (odds ratio (OR) 0.72, 95% confidence interval (CI) 0.56–0.94) and for sepsis (OR 0.66, 95% CI 0.47–0.93), but not for septic shock (OR 0.79, 95% CI 0.61–1.01) [19].

In 2013, a global collaborative cross-sectional study was conducted at 128 sites in 26 countries, including both the developed and the developing world (SPROUT study) [20]. This landmark study demonstrated that the prevalence of severe sepsis was 8.2% among children in ICU (<18 years old) with the associated hospital mortality of 25%, which was not different by age and between developed and developing countries [20]. Otherwise, this study also unveiled patient demographics, characteristics of infectious diseases and details of therapeutic interventions [20]. Furthermore, through the subgroup analysis of the SPROUT study comparing the patients in European PICUs with those in PICUs in the USA, PICU bed availability was suggested to affect the mortality of children with severe sepsis in the developed world [21] just like the findings in adult septic patients [22]. Table 1 shows the recent epidemiologic studies of pediatric sepsis from the developed world, including data from the Japanese PICUs [23] and the Italian PICUs [24].

**Table 1:** Epidemiology of pediatric sepsis in multicenter studies in developed countries since 2003

Authors, year	Country	Design Data source	Settings	Age	Incidence/prevalence	Mortality
Watson et al. 2003 [1]	USA 7 states	Retrospective Hospital discharge database	ICD-9^a^ coding & modified Angus criteria 1995	≤19 years	Incidence 0.56 per 1000 population (1995)	Hospital 10.3%
Hartman et al. 2013 [14]	USA 7 states	Retrospective Hospital discharge database	ICD-9^a^ coding & modified Angus criteria 1995, 2000, 2005	≤19 years	Incidence 0.89 per 1000 population (2005)	Hospital 8.9% (2005)
Ruth et al. 2014 [17]	USA 43 PICUs	Retrospective PHIS^b^ database	ICD-9^a^ coding & modified Angus criteria 2004–2012	≤18 years	Prevalence 6.2% (2004) to 7.7% (2012)	Hospital 14.4%; 18.9% (2004) to 12.0% (2012)
Wolfler et at. 2008 [23]	Italia 15 PICUs	Prospective	Proulx’s criteria^c^ 2004–2005	≤16 years	Incidence Severe sepsis 1.6% Septic shock 2.1%	ICU Severe sepsis 17.7% Septic shock^d^ 50.8%
Shime et al. 2011 [22]	Japan 9 PICUs	Prospective	Severe sepsis	≤15 years	Prevalence 1.4%	28-day 18.9%
Schlapbach et al. 2015 [18]	Australia/New Zealand 9 PICUs & 22 mixed ICUs	Retrospective ^d^ANZPIC registry	Invasive infection, sepsis, septic shock 2002–2013	<16 years	Prevalence Invasive infection 6.9% Sepsis 2.9% Septic shock 2.1%	ICU Invasive infection 3.9% Sepsis 5.6% Septic shock 17.0%
Weiss et al. 2015 (SPROUT) [19]	26 countries worldwide 128 PICUs	Point prevalence	Severe sepsis 5 days, 2013–2014	<18 years	Prevalence 8.2%	^e^ICU 23% ^e^Hospital 24%

^a^ *ICD-9* International Classification of Diseases, 9th Revision

^b^ *PHIS* Pediatric Health Information Systems

^c^Proulx’s definition of septic shock was different from Goldstein’s definition, now common in the world. Briefly, Proulx’s septic shock necessitated hypotension despite 20 mls/kg of fluid administration, while Goldstein’s septic shock is defined as inadequate perfusion, regardless of blood pressure, after 40 mls/kg of fluid resuscitation

^d^ *ANZPIC* Australian and New Zealand Paediatric Intensive Care

^e^These mortality rates were only for the sites in the developed world

These newly published epidemiologic researches also reported underlying conditions and infection sites. Hartman et al. reported a decreasing proportion of severe sepsis children with underlying comorbidities in 2005 compared with 2000 and 1995 (49.7% in 2005, 58.8% in 2000, and 63.3% in 1995). Neuromuscular, cardiovascular, and respiratory disorders were the most common comorbidities through all those years. Infection sites had been identified less frequently in 2005 than in 2000 and 1995 (54% in 2005, 74% in 2000, and 80% in 1995; *p* < 0.001), especially among neonates. Respiratory infection accounted for nearly half of all the identified cases (48.9% in 2005, 45.0% in 2000, and 47.1% in 1995), which was most frequent infection sites, followed by bacteremia (18.1% in 2005, 26.6% in 2000, and 20.7% in 1995) [15]. Ruth et al. revealed from their multicenter database that the proportion of severe sepsis children with at least one comorbidity had been increasing from 64.9% in 2002 to 76.6% in 2012 (*p* < 0.001), which was much higher than the previous national estimate in the USA (49.0% [1]), and that those children had a higher mortality rate than children without any comorbidity (15.8% vs. 10.4%, *p* < 0.001). After adjusting for age and organ dysfunction, children with malignancies were proved to have greater odds of mortality compared with those without (OR 1.93, 95% CI 1.79–2.08). Similarly, hematological/immunological disorders (OR 1.49, 95% CI 1.35–1.64) and cardiovascular conditions (OR 1.41, 95% CI 1.33–1.50) were found to be risks of mortality. Presumed sites of infection were noted in 91.5% of the patients, with bloodstream and respiratory tract most common (67.8 and 57.2%, respectively) [18]. Schlapbach et al. showed through multivariate analyses that the factors significantly associated with mortality in pediatric sepsis were oncological conditions (OR 1.95, 95% CI 1.41–2.69), bone marrow transplantation (OR 2.80, 95% CI 1.76–4.44), chronic neurological disorders (OR 1.76, 95% CI 1.23–2.52), chronic renal failure (OR 3.22, 95% CI 1.43–7.24), and the severity markers. The severity markers included implementation of mechanical ventilation in the first hour after PICU admission (OR 3.77, 95% CI 2.97–4.77), use of extracorporeal membrane oxygenation (OR 2.47, 95% CI 1.46–4.16) and renal replacement therapy (OR 4.68, 95% CI 3.43–6.40), and complication of acute respiratory distress syndrome (OR 1.53, 95% CI 1.01–2.32) [19]. Contrary to these findings, the SPROUT study revealed that the presence of any comorbidity did not significantly affect PICU mortality (*p* = 0.35). The mortality rate was, however, highest in children with solid organ/stem cell transplant (48.2%), followed by those with malignancies (41.3%), renal diseases (38.2%), and hematologic/immunologic conditions (37.7%). This study also showed the most common infection sites as respiratory tract (40%) and bloodstream (19%) [20].

It is speculated that the discrepancies of the epidemiologic data and risk factors between these studies might have originated from the differences in study population, diagnostic definition, and precision of the databases.

## Antimicrobials

**Fig. 1:**
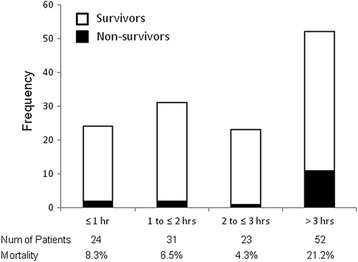
Time from sepsis recognition to initial antimicrobial administration with survival fraction. The shaded portion of each *bar* indicates the number of non-survivors in each time interval. Cited from reference [26]. (Promotional and commercial use of the material in print, digital, or mobile device format is prohibited without the permission from the publisher Wolters Kluwer. Please contact healthpermissions@wolterskluwer.com for further information.)

The early administration of antibiotics and hemodynamic stabilization with fluid resuscitation and inotropic/vasopressor support are like both wheels of a vehicle for the initial management of sepsis. Kumar et al. elegantly showed in their retrospective cohort study that the earlier administration of appropriate antibiotics was associated with higher survival rate for adult septic shock patients after the onset of persistent or recurrent hypotension [25]. In pediatric sepsis, Weiss et al. recently reported the similar results [26]. They retrospectively investigated 130 children with severe sepsis or septic shock treated in their PICU and found that more than 3-h delay of appropriate antibiotic administration after sepsis recognition was associated with a significant increase in PICU mortality (OR 3.92, 95% CI 1.27–12.06; Fig. 1) and fewer organ dysfunction-free days (16 vs. 20; *p* = 0.04). These associations persisted even after the adjustment of confounders [26]. However, it should be kept in mind that during the initial 3 h the delay in the first appropriate antibiotic administration did not lead to increased mortality [26], which was different from Kumar’s adult study [25].

## Hemodynamic management

### Early goal-directed therapy

**Fig. 2:**
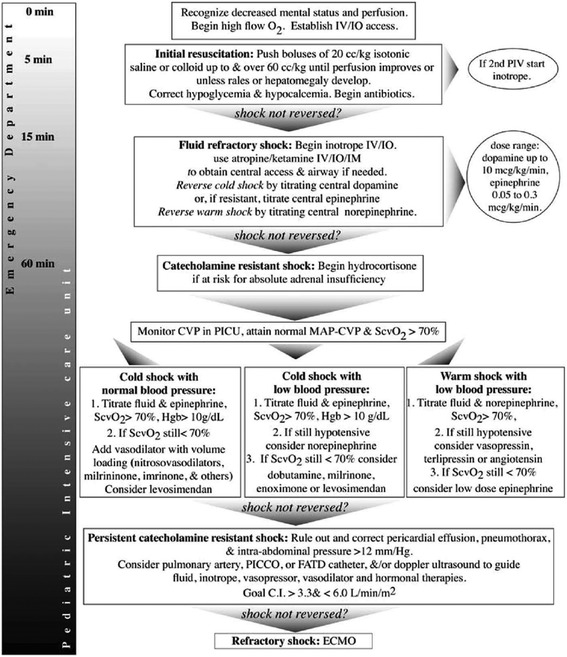
The American College of Critical Care Medicine-Pediatric Advanced Life Support (ACCM-PALS) algorithm. This algorithm aims at time sensitive, goal-directed stepwise management of hemodynamic support in infants and children, supported by the Surviving Sepsis Campaign guidelines 2012. Cited from reference [3]. (Promotional and commercial use of the material in print, digital or mobile device format is prohibited without the permission from the publisher Wolters Kluwer. Please contact healthpermissions@wolterskluwer.com for further information.)

Since Rivers et al. published so-called “early goal-directed therapy (EGDT)” with striking mortality reduction for septic shock adults (30.5% vs. 46.5%; *p* = 0.009) in 2001 [27], the cornerstone of the initial hemodynamic management for children with septic shock has also been aggressive fluid resuscitation and then inotropic/vasoactive support for fluid-refractory shock patients [2, 28, 29]. De Oliveira et al. investigated the American College of Critical Care Medicine-Pediatric Advanced Life Support (ACCM-PALS) algorithm (Fig. 2), including continuous S_CV_O_2_ monitoring and red blood cell transfusion similar to the Rivers’ original EGDT, for 102 children with severe sepsis or fluid-refractory septic shock (1 month to 18 years of age) in a randomized controlled trial fashion and reported the improved survival in the intervention group (28-day mortality 11.8% vs. 39.2%, *p* = 0.002) [30]. Sankar et al. also suggested that even the intermittent measurement of S_CV_O_2_, compared with no S_CV_O_2_ monitoring, could contribute to increased survival through their prospective cohort study with 120 children with fluid-refractory septic shock (<17 years of age) [31].

Nevertheless, for the past few years, 3 multicenter randomized controlled trials (ProCESS [32], ARISE [33], and ProMISe [34]) and one meta-analysis [35] demonstrated that the standard hemodynamic management without continuous S_CV_O_2_ targeting was equally effective to the EGDT for septic shock adults. Considering the recent trend in adult critical care, an original form of EGDT will not be applied to pediatric sepsis any longer.

### Hemodynamic evaluation and monitoring

As for substitute monitoring, lactate clearance was suggested to be non-inferior to S_CV_O_2_ monitoring to evaluate the reversal of tissue hypoxia in septic shock adults [36, 37], which is now a part of the Surviving Sepsis Campaign bundles since 2012 [3]. This might also be the case with septic children. With their prospective cohort of 77 children with severe sepsis (<18 years of age), Scott et al. recently demonstrated that the patients whose serum lactate was normalized (<2 mmol/L) within 2–4 h of the initial measurement had a significantly lower risk of persistent organ dysfunction over 48 h (relative risk (RR) 0.46, 95% CI 0.29–0.73) [38]. On the other hand, the patients who only achieved lactate clearance by more than 10% of the initial level did not show a significant reduction of organ dysfunction [38].

Otherwise, transthoracic echocardiography has been attracting more attention as a noninvasive tool to repeatedly evaluate hemodynamics among septic children as sepsis-associated myocardial dysfunction is known more widely [39, 40]. Sankar et al. pointed out that the prevalence of left ventricular diastolic dysfunction among 56 children with fluid-refractory septic shock (3 months to 17 years of age) was as much as 41% with their mortality rate of 43% [41]. Raj et al. also investigated 30 septic shock children and adolescents (1 month to 21 years of age) and showed that the prevalence of left ventricular systolic, diastolic, and both dysfunction was 37%, 33%, and 17%, respectively, [42]. Furthermore, Abdel-Hady et al. suggested with their 20 full-term neonatal cohort with sepsis that the use of a tissue Doppler imaging would more sensitively detect myocardial dysfunction missed by conventional echocardiography [43]. Basu et al. also showed the efficacy of strain echocardiography to detect impaired myocardial performance despite normal ejection fraction and fractional shortening among children with septic shock (1–13 years of age) [44].

With all these findings of great interest, unfortunately, clinical relevance of echocardiography-guided management, especially to prognosis, has not been fully studied yet. Ranjit et al. suggested from their prospective cohort of 48 children with septic shock (1 month to 16 years of age) the efficacy of bedside echocardiography along with arterial pressure monitoring to recognize sepsis-associated myocardial dysfunction and uncorrected hypovolemia and then titrate fluid and inotropes/vasopressors [45]. Haileselassie et al. recently reported from their retrospective cohort study of 23 children with sepsis (<19 years old) in their PICU, compared with their internal controls, that the septic patients had significantly worse strain, both longitudinal and circumferential, which was correlated with higher lactate levels but was not associated with ICU length of stay [46]. These studies have an inevitable risk of bias, so better-designed larger studies are essential to establish the effectiveness of echocardiography-guided hemodynamic management in pediatric sepsis.

### Fluid responsiveness

In adult critical care, fluid responsiveness has been regarded as more important than preload itself (i.e., intravascular volume or ventricular end-diastolic volume) in order to predict the efficacy of bolus fluid with avoiding fluid overload. Several dynamic parameters are promising to evaluate fluid responsiveness, such as systolic pressure variation (SPV), pulse pressure variation (PPV), and stroke volume variation (SVV), for ventilated adults. All of them derive from the analysis of variation in arterial pressure waveform caused by mechanical ventilation cycling. The recently published SSCG 2016 also suggested the usefulness of dynamic indices to evaluate fluid requirement for adult patients with sepsis [4]. On the other hand, Gan et al. conducted a systematic review of various static and dynamic indices to assess fluid responsiveness among ventilated children through the comparison of the areas under the receiver operating characteristic (ROC) curve [47]. They demonstrated that almost all the static indices were not helpful to predict fluid responsiveness, including heart rate, systolic arterial blood pressure, and central venous pressure. Moreover, disappointingly, most of the dynamic indices, including those based on arterial waveform (i.e., SPV, PPV, and SVV), inferior vena cava diameter and plethysmograph, also lacked predictive value, which was inconsistent with the findings in adults. The only reliable parameter was respiratory variation in aortic blood flow peak velocity (ΔV_peak_) measured with Doppler echocardiography, which predicted an increase in stroke volume by more than 15% with 10 mls/kg of bolus fluid. The authors speculated that the difference in efficacy of dynamic variables might be affected by higher chest wall and lung compliance, more compliant arterial vasculature, and lower cardiac ventricular compliance in children compared with adults [47]. The reliability of ΔV_peak_ to predict fluid responsiveness was also confirmed among mechanically ventilated children in another systematic review by Desgranges et al. [48]. Unfortunately, so far, the optimal cutoff value of ΔV_peak_ has not been identified to discriminate responders from non-responders because those values ranged from 7 to 20% across six included studies [48].

Passive leg raising (PLR) is a simple maneuver to assess fluid responsiveness, “virtual” fluid challenge by facilitating venous return from lower extremities. Three well-conducted systematic reviews have recently proved PLR to be highly valid to predict fluid responsiveness in adults with circulatory failure, whether they are mechanically ventilated or spontaneously breathing [49–51]. In addition, when PLR was conducted, changes in such variables as cardiac output, stroke volume, and aortic blood flow predicted fluid responsiveness more accurately than those in pulse pressure [50, 51]. On the other hand, PLR has ever been tested only in one study for children [52]. Lukito et al. recruited 40 children (1–8 years old) in their PICU with diverse diagnoses, either mechanically ventilated or spontaneously breathing, and showed that an increase in cardiac index after PLR maneuver was significantly associated with fluid responsiveness [52].

It should be noted that children with sepsis accounted for a limited percentage of the participants in these studies [47, 48, 52], so the significance of these dynamic indices has not been clarified yet in the hemodynamic management of pediatric sepsis. Moreover, in both adult and pediatric critical care, the concept that these dynamic indices are more predictable of fluid responsiveness than static hemodynamic parameters would be unchallenged any longer, but it must be taken into consideration that hemodynamic management based on these dynamic indices has never been validated to improve patients’ clinical outcomes so far.

### Fluid management

From the viewpoint of fluid resuscitation, the FEAST trial [53], the largest randomized controlled trial (RCT) of fluid bolus therapy for 3141 sub-Saharan children with severe infection and impaired perfusion (60 days to 12 years of age), aroused much controversy since its publication in 2011. Contrary to expectations, the trial revealed that the patients who were given any bolus fluid, saline or 5% albumin, as a part of initial intervention had a significantly higher 48-h mortality rate than those not given bolus fluid (RR 1.45; 95% CI, 1.13–1.86) [53]. This shocking results were plausibly explained due to a high prevalence of malaria (57%) or severe anemia (hemoglobin <5 g/dL, 32%) among the study population and under-recognition of fluid overload [54]. However, their post hoc analyses of excess mortality mechanism [55] as well as their prespecified subgroup analyses [53] contradicted those rationalizations. More reasonable explanation for adverse outcomes in the bolus group would be rapid reduction in sympathetically mediated circulatory compensation, involvement of ischemia-reperfusion injury or lack of advanced monitoring, mechanical ventilation, and inotropic/vasoactive supports in the resource-limited settings after the initial fluid resuscitation [56–59]. The subsequently conducted systematic review of fluid bolus therapy in pediatric sepsis was affected by a huge impact of the FEAST trial [60], which found a harmful effect of fluid boluses. More recently, Gelbart et al. systematically reviewed the studies of fluid bolus therapy in hospitalized children with severe sepsis or septic shock (29 days to 18 years of age), excluding those of lone tropical pathogens, such as malaria and dengue fever [61]. They found only three RCTs, two from India and the other from Brazil [30], and 8 observational studies, largely retrospective. Unfortunately, those studies were too heterogeneous in methodology and findings all with small sample size, which precluded meta-analysis [61].

When it comes to a type of resuscitation fluid, the SSCG 2012 did not indicate the superiority of either isotonic crystalloids or colloids [3]. However, the equivalency of isotonic crystalloids to colloids in survival was only based on the three RCTs in children with dengue shock syndrome [62–64], which was obviously different from the situation in developed countries. In the FEAST trial, which included a large proportion of children with malaria, there was no difference in 48-h and 4-week mortality between albumin-bolus and saline-bolus groups (10.6% vs. 10.5% for 48-h mortality, RR 1.00, 95% CI 0.78–1.29; 12.2% vs. 12.0% for 4-week mortality, RR 1.01, 95% CI 0.80–1.28). As described above, both of the bolus groups had significantly higher mortality than the no bolus group [53], but again, this is quite a different setting from developed countries. Jian et al. recently published a meta-analysis of RCTs in which albumin vs. other fluids was compared for fluid resuscitation in various populations with sepsis. They demonstrated no significant effect of albumin over other types of fluid on all-cause mortality among children with sepsis (fixed effect model, RR 0.92, 95% CI 0.74–1.14; random effect model, RR 0.55, 95% CI 0.21–1.45) [65]. Unfortunately, however, it must be kept in mind that all the RCTs adopted in this meta-analysis were also conducted in developing countries with a high percentage of malaria patients, for whom the FEAST trial [53] largely accounted [65]. On the other hand, the SPROUT study identified albumin use as a significant risk factor of PICU mortality adjusted for age, sex, severity score, geographic region, and number of comorbidities (adjusted OR 2.50, 95% CI 1.54–4.05) [20]. These negative findings of albumin use in children with sepsis are in contrast to the non-significant but more favorable findings in adults with sepsis [4, 66]. Otherwise, it would be recommended to avoid synthetic colloids for children in terms of renal insult as well, based on the findings [67–69] and recommendation [4] in adult sepsis.

Considering these facts, so far, it is very difficult to show any recommendation or suggestion regarding the optimal dose and type of resuscitation fluid in the management of pediatric sepsis. However, at least in developed countries, it would be reasonable to continue current practice suggested in the SSCG 2012 [3] as well as judicious use of albumin. Well-designed pragmatic RCTs are definitely needed to clarify the optimal type and dose of resuscitation fluid in pediatric sepsis in advanced countries.

Excessively positive fluid balance has been pointed out to be associated with poor outcomes for both critically ill adults and children [70]. However, in their case-control study with the multicenter pediatric septic shock registry in the USA (10 years old or younger), Abulebda et al. suggested that both fluid balance for the first 24 h and cumulative percent positive fluid balance for 7 days after PICU admission were not associated with mortality or multiorgan dysfunction in the intermediate- and high-risk group [71], stratified with the newly devised pediatric sepsis biomarker risk model [72].

### Inotropic/vasoactive agents

In terms of inotropic/vasoactive agents for septic shock, noradrenaline has recently been regarded as the first line for adults mainly because of fewer arrhythmic events [3, 73, 74]. In pediatric septic shock, comparative studies were lacking at the publication of the SSCG 2012 [3], which did not specify any inotropic/vasoactive agent. Fortunately, for the past couple of years, two RCTs were published to compare dopamine with adrenaline as the first line agent [75, 76]. Ventura et al. randomly assigned 120 children with fluid-refractory septic shock (1 month to 15 years of age) to receive either continuous infusion of dopamine or adrenaline [75]. Study drugs were waxed up every 20 min (dopamine 5, 7.5, 10 μg/kg/min vs. adrenaline 0.1, 0.2, 0.3 μg/kg/min) to achieve the predetermined hemodynamic stabilization criteria and after the maximum dose another catecholamine could be replaced at physicians’ discretion. The primary outcome was the 28-day mortality, which was significantly lower in the adrenaline group than in the dopamine group (7% vs. 21%, *p* = 0.033). In addition, dopamine was associated with death (OR, 6.5; 95% CI, 1.1–37.8) and healthcare–associated infection (OR, 67.7; 95% CI, 5.0–910.8) in the multivariate analysis [75]. Narayanan et al. also conducted a pilot RCT, in which they compared dopamine with adrenaline as the first line in a different regimen (dopamine 10, 15, 20 μg/kg/min vs. adrenaline 0.1, 0.2, 0.3 μg/kg/min every 10 min) among 60 fluid-refractory hypotensive “cold” septic shock children (3 month to 12 years of age). The primary outcome was the rate of shock resolution within the first hour of resuscitation, which was significantly higher in the adrenaline group (41% vs. 13%, *p* = 0.019), but the mortality was not significantly different (48% in the adrenaline group vs. 58% in the dopamine groups, *p* = 0.605) [76]. These RCTs may apparently suggest that adrenaline is replacing dopamine as the first line in the management of pediatric septic shock, but it would be better to say that they just compared two regimens of hemodynamic management rather than the two agents. Indeed, Deep et al. revealed the two distinct hemodynamic patterns on presentation among 36 prospectively registered children excluding neonates with fluid-refractory septic shock [77]. In general, most of the children with community-acquired septic shock presented in “cold shock,” while all the children with hospital-acquired septic shock manifested “warm shock”. However, some of the patients in “cold shock” who were initially commenced on adrenaline required noradrenaline or were switched onto milrinone later, while some of the patients in “warm shock” who initially responded to noradrenaline subsequently developed low cardiac output and required adrenaline [77]. Considering these facts, the universal application of a single agent as the first line might be hazardous and the subsequent meticulous optimization of hemodynamic support would be inevitable for the management of septic shock children.

In the case of adult septic shock with catecholamine-resistant vasodilatory hypotension, vasopressin, and its long-acting analog terlipressin have been suggested as an alternative to restore optimal perfusion pressure since the SSCG 2012 [3, 78]. On the other hand, the use of these agents was not supported in children for lack of overt clinical benefits [3, 79, 80]. More recently, Masarwa et al. published a systematic review comparing vasopressin and terlipressin with conventional treatment in children (0–18 years old) with refractory shock of all causes [81]. They cited 3 RCTs [79, 80, 82] and found no association between the use of vasopressin/terlipressin and mortality (RR 1.19; 95% CI 0.71–2.00; *I* ^2^ = 28%). They also pointed out a non-significant but concerning tendency toward more tissue ischemia in patients treated with vasopressin/terlipressin (RR 1.48; 95% CI 0.47–4.62; *I* ^2^ = 0%) [81]. This difference in efficacy may possibly originate from variable levels of intrinsic vasopressin and copeptin in children with septic shock, contrary to relative vasopressin deficiency among adult patients [83]. Otherwise, methylene blue was also suggested as another vasoconstrictor for catecholamine-resistant vasodilatory shock [84], which has not been adequately evaluated yet.

## Adjunct therapies

In the pediatric considerations in the SSCG 2012, the timely supplementation of hydrocortisone was suggested for children with fluid-refractory catecholamine-resistant septic shock and suspected or proven absolute (classic) adrenal insufficiency, which was rated as grade 1A, i.e., strong recommendation with high-quality evidence [3, 85, 86], even without adequately powered trials. On the other hand, when it comes to the efficacy of corticosteroids in the more common situation of critical illness-related corticosteroid insufficiency in pediatric septic shock, well-designed research is desperately scarce [85, 86]. Menon et al. recently conducted a systematic review of the RCTs, only to find out that most of them were published regarding dengue shock before 1996 in developing countries [87]. Their meta-analysis showed no survival benefit in those who received corticosteroids compared with those who did not [87]. Furthermore, in the SPROUT study, the use of corticosteroids was significantly associated with mortality in the multivariate analysis (adjusted OR 1.58, 95% CI 1.01–2.49) [20]. Despite lack of convincing evidence, a recent Canadian national survey revealed that almost all the pediatric intensivists (91.4%) would administer corticosteroids to patients in a persistent shock who had received 60 mL/kg of fluid and were on two or more vasoactive medications [88]. In that survey, more than 80% of the respondents stated that they were also willing to recruit their persistent shock patients into future RCTs, but at the same time, they answered that many of them would prescribe open-label corticosteroids [88] if their patients deteriorated, which implies potential difficulties of conducting an effective RCT [89]. Deducing a pile of available research findings [90–95] and the suggestion in the SSCG 2012 for adults [3], it would remain reasonable to consider the administration of low-dose hydrocortisone only to children with fluid-refractory, catecholamine-resistant septic shock. Well-designed, large-scale RCTs are definitely needed to evaluate the efficacy of corticosteroids in pediatric septic shock with a pragmatic target population, specific inclusion/exclusion criteria, adverse event reporting, and realistic endpoints [89] (Table 2).

**Table 2:** The summary of the newly added findings on the management of pediatric sepsis

# Administer the first appropriate antimicrobials within 3 h after the recognition	
# Lactate clearance might be promising for children with elevated lactate level	
# Transthoracic echocardiography should be encouraged to use for the evaluation of the hemodynamics and treatment response	
# Dynamic variables would be more preferable to static variables to evaluate fluid responsiveness, but the only reliable parameter is respiratory variation in aortic blood flow peak velocity (ΔV_peak_) measured with echocardiography so far	
# Adrenaline would be more preferable to dopamine for the first line catecholamine in children with fluid-refractory septic shock	
# Serial meticulous evaluation of the hemodynamics and adjustment of the management are essential	
# Be more cautious about the use of vasopressin/terlipressin for children with fluid-refractory septic shock	
# Be more “conservative” than ever about the administration of corticosteroids	

With other adjunct therapies, the efficacy of extracorporeal therapies for pediatric sepsis, including extracorporeal life support (ECLS), renal replacement therapy (RRT), and plasma exchange (PE), has been investigated these few years. The consideration of ECLS was suggested in the SSCG 2012 for refractory septic shock children as a last resort of hemodynamic management [3], but this suggestion is based only on experience in a limited number of centers [96–98]. More recently, Ruth et al. demonstrated from the multicenter PICU database in the USA the increasing utilization of ECLS for septic children (3.6% in 2004–2008 vs. 4.0% in 2009–2012 among all with severe sepsis), especially for those with three or more organ dysfunctions from 2004 to 2012 (6.9–10.3%). They also reported that the mortality rate was 47.8% with the trend of gradual declining among the children who underwent ECLS [99]. Smith et al. lately reported their experience of ECLS runs for 9 children with neutropenic sepsis as 44% survival, which was previously contraindicated because of pessimistic prognosis [100]. As for RRT, Ruth et al. revealed in their multicenter PICU database that RRT was applied to 19.0% of septic children (0–18 years old), but the utilization of RRT had been significantly decreasing from 2004 to 2012. The associated mortality rate was 32.3% with RRT alone and 58.0% with both ECLS and RRT [99]. The SSCG 2012 suggested consideration of the use of diuretics and RRT to avoid greater than 10% total body weight fluid overload [3] based mainly on a single-center retrospective study [101]. Unfortunately, this suggestion, especially the threshold value of fluid overload, has not been validated well among septic children yet [102–104]. The efficacy of PE in pediatric sepsis is also ambiguous. Kawai et al. suggested the possible efficacy of PE at an early stage for their 14 children on ECLS in terms of the recovery of organ dysfunctions and hemodynamic status [105], while meta-analysis conducted by Rimmer et al. demonstrated no survival benefit of PE in septic children (*n* = 66, RR 0.96, 95% CI 0.28–3.38) [106]. Unfortunately, this meta-analysis was under-powered.

## “Sepsis bundle” approach (ACCM/PALS algorithm)

In the SSCG 2012, the initial management bundle is advocated to improve the performance quality of adult sepsis care [3]. The recently conducted global prospective observational study (IMPreSS study) demonstrated the survival benefit of compliance with the bundled approach for adults with severe sepsis or septic shock [107]. In pediatric sepsis, the SSCG panel continued to recommend compliance with the ACCM-PALS algorithm for the initial management of septic shock (Fig. 2) [29] from 2008 through 2012 [2, 3]. This algorithm had been proved effective in a few studies [30, 108–110], and its adherence in the clinical settings has recently been investigated more vigorously. Paul et al. prospectively investigated in their emergency department adherence to the five algorithmic time-specific goals; early recognition, vascular access, intravenous fluids up to 60 mls/kg, vasopressors for fluid-refractory shock, and antibiotic administration [111]. They found out low adherence rate to the total algorithm, only 19%, as well as to adequate fluid resuscitation and timely vasopressor start, 37 and 35%, respectively. They also revealed a significantly shorter hospital length of stay among the adherence group compared with that among the non-adherence (6.8 vs. 10.9 days, *p* = 0.009) [111]. They subsequently commenced quality improvement initiatives for higher adherence to the ACCM-PALS algorithm, especially focusing on the timely fluid resuscitation up to 60 mls/kg within 60 min [112]. With their vigorous intervention to the ED staff, adherence to fluids, vasoactive agents, and the total bundle all improved and finally reached 100% and remained nearly 100% thereafter [112]. Long et al. prospectively conducted the same sort of quality improvement intervention study, focusing on venous blood gas sampling, timely fluid resuscitation, and antibiotic administration [113]. They achieved the significant reduction of time to intravenous access, antibiotic administration, and fluid administration, and more importantly, significantly shorter hospital length of stay (96 h in pre-intervention vs. 80 h in post-intervention; hazard ratio 1.36, 95% CI 1.04–1.80) [113]. A couple of other retrospective cohort studies suggested the beneficial effect of protocolized initial management for reduced complication rate of acute kidney injury [114] and some organ dysfunctions [115]. It should be noted that all these studies are of single-center, before-after or retrospective design, but every effort to comply with “sepsis bundles” is highly likely to improve performance in the management of septic children.

## Post-intensive care sequelae

As mentioned above, morality of pediatric sepsis seems to be gradually declining for the past decade. On the other hand, long-term sequelae among pediatric sepsis survivors have not been well investigated yet.

The post hoc analysis of the RESOLVE trial [116] revealed that as much as 34% of the 28-day pediatric sepsis survivors who required both vasoactive agents and mechanical ventilation (38 weeks corrected gestation to 17 years of age at recruitment) had some decline in their functional status with 18% of at least moderate disability [117]. They also found out the risk factors associated with poor functional outcome; central nervous system and intra-abdominal infection sources, recent trauma, receipt of cardiopulmonary resuscitation prior to enrollment, and high severity index [117]. The SPROUT study revealed that as much as 17% of pediatric survivors through severe sepsis were complicated by at least moderate disabilities, while 28% at least mild disabilities [20].

Aspesberro et al. recently conducted a focused review of the literature regarding health-related quality of life (HRQoL) among pediatric critical care survivors (0–18 years old) [118]. They identified sepsis on ICU admission as one of the key determinants of poor HRQoL. They also found out low scores of behavioral and emotional measurement scales among meningococcal septic shock survivors and reduced aspects of neuropsychologic function among children with meningoencephalitis and sepsis [118]. Obviously, children who have survived sepsis are struggling for their premorbid performance.

## Conclusions

Comparing with the “pre-SSCG” era, more evidence has been accumulating in pediatric sepsis for the past decade. Above all, the SPROUT study has provided important implications about future research on pediatric sepsis based on a global epidemiologic data [20, 119]. Mortality seems to be declining gradually, thanks to wider acceptance of the SSCG, though the trend has not been firmly verified yet. Pediatric intensivists must keep it in mind that all the sepsis survivors cannot restore the premorbid level performance [120]. It would be desirable that multidisciplinary longitudinal follow-up should be coordinated for pediatric sepsis survivors. In addition, future clinical research for children with sepsis should adopt as outcome measures not only mortality but also long-term HRQoL to fully evaluate the impact of sepsis on children’s life.

## Abbreviations

ACCM: American College of Critical Care Medicine
CI: Confidence interval
ECLS: Extracorporeal life support
EGDT: Early goal-directed therapy
HRQoL: Health-related quality of life
ICD-9: The International Classification of Diseases, 9th edition, Clinical Modification
ICU: Intensive care unit
OR: Odds ratio
PALS: Pediatric Advanced Life Support
PE: Plasma exchange
PICU: Pediatric intensive care unit
PLR: Passive leg raising
PPV: Pulse pressure variation
RCT: Randomized controlled trial
ROC curve: Receiver operating characteristic curve
RR: Relative risk
RRT: Renal replacement therapy
S_CV_O_2_: Central venous oxygen saturation
SIRS: Systemic inflammatory response syndrome
SOFA: Sequential Organ Failure Assessment
SPV: Systolic pressure variation
SSCG: Surviving Sepsis Campaign guidelines
SVV: Stroke volume variation
ΔV_peak_: Respiratory variation in aortic blood flow peak velocity

## References

[1] Watson RS, Carcillo JA, Linde-Zwirble WT (2003). The epidemiology of severe sepsis in children in the United States. Am J Respir Crit Care Med.

[2] Dellinger RP, Levy MM, Carlet JM (2008). Surviving Sepsis Campaign: international guidelines for management of severe sepsis and septic shock: 2008. Intensive Care Med.

[3] Dellinger RP, Levy MM, Rhodes A (2013). Surviving sepsis campaign: international guidelines for management of severe sepsis and septic shock: 2012. Crit Care Med.

[4] Rhodes A, Evans LE, Alhazzani W (2017). Surviving sepsis campaign: international guidelines for management of sepsis and septic shock: 2016. Crit Care Med.

[5] Committee M of the AC of CP of CCMCC (1992). American College of Chest Physicians/Society of Critical Care Medicine Consensus Conference: definitions for sepsis and organ failure and guidelines for the use of innovative therapies in sepsis. Crit Care Med.

[6] Levy MM, Fink MP, Marshall JC (2003). 2001 SCCM/ESICM/ACCP/ATS/SIS International Sepsis Definitions Conference. Crit Care Med.

[7] Goldstein B, Giroir B, Randolph A (2005). International pediatric sepsis consensus conference: definitions for sepsis and organ dysfunction in pediatrics. Pediatr Crit Care Med.

[8] Churpek MM, Zadravecz FJ, Winslow C (2015). Incidence and prognostic value of the systemic inflammatory response syndrome and organ dysfunctions in ward patients. Am J Respir Crit Care Med.

[9] Kaukonen K-M, Bailey M, Pilcher D (2015). Systemic inflammatory response syndrome criteria in defining severe sepsis. N Engl J Med.

[10] Singer M, Deutschman CS, Seymour CW (2016). The third international consensus definitions for sepsis and septic shock (sepsis-3). JAMA.

[11] Weiss SL, Parker B, Bullock ME, et al. Defining pediatric sepsis by different criteria: discrepancies in populations and implications for clinical practice. Pediatr Crit Care Med. 2012;13(4):e219–26. 10.1097/PCC.0b013e31823c98da 22460773

[12] Weiss SL, Fitzgerald JC, Maffei FA (2015). Discordant identification of pediatric severe sepsis by research and clinical definitions in the SPROUT international point prevalence study. Crit Care.

[13] Piva JP, Garcia PCR (2016). Sepsis: from the stone age to nowadays without a precise definition. Pediatr Crit Care Med.

[14] Shime N, Kawasaki T, Nakagawa S (2017). Proposal of a new pediatric sequential organ failure assessment score for possible validation. Pediatr Crit Care Med.

[15] Hartman ME, Linde-Zwirble WT, Angus DC (2013). Trends in the epidemiology of pediatric severe sepsis. Pediatr Crit Care Med.

[16] Balamuth F, Weiss SL, Neuman MI (2014). Pediatric severe sepsis in U.S. children’s hospitals. Pediatr Crit Care Med.

[17] Angus DC, Linde-Zwirble WT, Lidicker J (2001). Epidemiology of severe sepsis in the United States: analysis of incidence, outcome, and associated costs of care. Crit Care Med.

[18] Ruth A, McCracken CE, Fortenberry JD (2014). Pediatric severe sepsis: current trends and outcomes from the pediatric health information systems database. Pediatr Crit Care Med.

[19] Schlapbach LJ, Straney L, Alexander J (2015). Mortality related to invasive infections, sepsis, and septic shock in critically ill children in Australia and New Zealand, 2002–13: a multicentre retrospective cohort study. Lancet Infect Dis.

[20] Weiss SL, Fitzgerald JC, Pappachan J (2015). Global epidemiology of pediatric severe sepsis: the sepsis prevalence, outcomes, and therapies study. Am J Respir Crit Care Med.

[21] Jr JSG, Markovitz BP, Brierley J (2016). Comparison of pediatric severe sepsis managed in U.S. and European ICUs. Pediatr Crit Care Med.

[22] Levy MM, Artigas A, Phillips GS (2012). Outcomes of the Surviving Sepsis Campaign in intensive care units in the USA and Europe: a prospective cohort study. Lancet Infect Dis.

[23] Shime N, Kawasaki T, Saito O (2012). Incidence and risk factors for mortality in paediatric severe sepsis: results from the national paediatric intensive care registry in Japan. Intensive Care Med.

[24] Wolfler A, Silvani P, Musicco M (2008). Incidence of and mortality due to sepsis, severe sepsis and septic shock in Italian Pediatric Intensive Care Units: a prospective national survey. Intensive Care Med.

[25] Kumar A, Roberts D, Wood KE (2006). Duration of hypotension before initiation of effective antimicrobial therapy is the critical determinant of survival in human septic shock. Crit Care Med.

[26] Weiss SL, Fitzgerald JC, Balamuth F (2014). Delayed antimicrobial therapy increases mortality and organ dysfunction duration in pediatric sepsis. Crit Care Med.

[27] Rivers E, Nguyen B, Havstad S (2001). Early goal-directed therapy in the treatment of severe sepsis and septic shock. N Engl J Med.

[28] Carcillo JA, Fields AI (2002). Clinical practice parameters for hemodynamic support of pediatric and neonatal patients in septic shock. Crit Care Med.

[29] Brierley J, Carcillo JA, Choong K (2009). Clinical practice parameters for hemodynamic support of pediatric and neonatal septic shock: 2007 update from the American College of Critical Care Medicine. Crit Care Med.

[30] de Oliveira CF, de Oliveira DSF, Gottschald AFC (2008). ACCM/PALS haemodynamic support guidelines for paediatric septic shock: an outcomes comparison with and without monitoring central venous oxygen saturation. Intensive Care Med.

[31] Sankar J, Sankar MJ, Suresh CP (2014). Early goal-directed therapy in pediatric septic shock: comparison of outcomes “with” and “without” intermittent superior venacaval oxygen saturation monitoring: a prospective cohort study. Pediatr Crit Care Med.

[32] Yealy DM, Kellum JA, Huang DT (2014). A randomized trial of protocol-based care for early septic shock. N Engl J Med.

[33] Bailey M, Bellomo R, Peter A (2014). Goal-directed resuscitation for patients with early septic shock. N Engl J Med.

[34] Mouncey PR, Osborn TM, Power GS (2015). Trial of early, goal-directed resuscitation for septic shock. N Engl J Med.

[35] Angus DC, Barnato AE, Bell D (2015). A systematic review and meta-analysis of early goal-directed therapy for septic shock: the ARISE, ProCESS and ProMISe Investigators. Intensive Care Med.

[36] Nguyen HB, Rivers EP, Knoblich BP (2004). Early lactate clearance is associated with improved outcome in severe sepsis and septic shock. Crit Care Med.

[37] Jones AE, Shapiro NI, Trzeciak S (2010). Lactate clearance vs central venous oxygen saturation as goals of early sepsis therapy. JAMA.

[38] Scott HF, Brou L, Deakyne SJ (2016). Lactate clearance and normalization and prolonged organ dysfunction in pediatric sepsis. J Pediatr.

[39] Rudiger A, Singer M (2007). Mechanisms of sepsis-induced cardiac dysfunction. Crit Care Med.

[40] Zanotti-Cavazzoni SL, Hollenberg SM (2009). Cardiac dysfunction in severe sepsis and septic shock. Curr Opin Crit Care.

[41] Sankar J, Das RR, Jain A (2014). Prevalence and outcome of diastolic dysfunction in children with fluid refractory septic shock—a prospective observational study. Pediatr Crit Care Med.

[42] Raj S, Killinger JS, Gonzalez JA (2014). Myocardial dysfunction in pediatric septic shock. J Pediatr.

[43] Abdel-Hady HE, Matter MK, El-Arman MM (2012). Myocardial dysfunction in neonatal sepsis: a tissue Doppler imaging study. Pediatr Crit Care Med.

[44] Basu S, Frank LH, Fenton KE (2012). Two-dimensional speckle tracking imaging detects impaired myocardial performance in children with septic shock, not recognized by conventional echocardiography. Pediatr Crit Care Med.

[45] Ranjit S, Aram G, Kissoon N (2014). Multimodal monitoring for hemodynamic categorization and management of pediatric septic shock. Pediatr Crit Care Med.

[46] Haileselassie B, Su E, Pozios I (2016). Strain echocardiography parameters correlate with disease severity in children and infants with sepsis. Pediatr Crit Care Med.

[47] Gan H, Cannesson M, Chandler JR (2013). Predicting fluid responsiveness in children: a systematic review. Anesth Analg.

[48] Desgranges FP, Desebbe O, Pereira de Souza Neto E (2016). Respiratory variation in aortic blood flow peak velocity to predict fluid responsiveness in mechanically ventilated children: a systematic review and meta-analysis. Paediatr Anaesth.

[49] Bentzer P, Griesdale DE, Boyd J (2016). Will this hemodynamically unstable patient respond to a bolus of intravenous fluids?. JAMA.

[50] Cherpanath TGV, Hirsch A, Geerts BF (2016). Predicting fluid responsiveness by passive Leg raising: a systematic review and meta-analysis of 23 clinical trials. Crit Care Med.

[51] Monnet X, Marik P, Teboul JL (2016). Passive leg raising for predicting fluid responsiveness: a systematic review and meta-analysis. Intensive Care Med.

[52] Lukito V, Djer MM, Pudjiadi AH (2012). The role of passive leg raising to predict fluid responsiveness in pediatric intensive care unit patients. Pediatr Crit Care Med.

[53] Maitland K, Kiguli S, Opoka RO (2011). Mortality after fluid bolus in African children with severe infection. N Engl J Med.

[54] Maitland K, Akech SO, Russell EC (2011). Mortality after fluid bolus in African children with sepsis. N Engl J Med.

[55] Maitland K, George EC, Evans JA (2013). Exploring mechanisms of excess mortality with early fluid resuscitation: insights from the FEAST trial. BMC Med.

[56] Duke T (2011). What the African fluid-bolus trial means. Lancet.

[57] Hilton AK, Bellomo R (2011). Totem and taboo: fluids in sepsis. Crit Care.

[58] Hilton AK, Bellomo R (2012). A critique of fluid bolus resuscitation in severe sepsis. Crit Care.

[59] Myburgh J, Finfer S (2013). Causes of death after fluid bolus resuscitation: new insights from FEAST. BMC Med.

[60] Ford N, Hargreaves S, Shanks L. Mortality after fluid bolus in children with shock due to sepsis or severe infection: a systematic review and meta-analysis. PLoS One. 2012;7(8):e43953. 10.1371/journal.pone.0043953 PMC3431361 22952819

[61] Gelbart B, Glassford NJ, Bellomo R (2015). Fluid bolus therapy-based resuscitation for severe sepsis in hospitalized children. Pediatr Crit Care Med.

[62] Dung NM, Day NP, Tam DT (1999). Fluid replacement in dengue shock syndrome: a randomized, double-blind comparison of four intravenous-fluid regimens. Clin Infect Dis.

[63] Ngo NT, Cao XT, Kneen R (2001). Acute management of dengue shock syndrome: a randomized double-blind comparison of 4 intravenous fluid regimens in the first hour. Clin Infect Dis.

[64] Wills B, Dung N, Loan H (2005). Comparison of three fluid solutions for resuscitation in dengue shock syndrome. N Engl J Med.

[65] Jiang L, Jiang S, Zhang M (2014). Albumin versus other fluids for fluid resuscitation in patients with sepsis: a meta-analysis. PLoS One.

[66] Delaney AP, Dan A, Mccaffrey J (2011). The role of albumin as a resuscitation fluid for patients with sepsis: a systematic review and meta-analysis. Crit Care Med.

[67] Myburgh JA, Finfer S, Bellomo R (2012). Hydroxyethyl starch or saline for fluid resuscitation in intensive care. N Engl J Med.

[68] Perner A, Haase N, Guttormsen AB (2012). Hydroxyethyl starch 130/0.42 versus Ringer’s acetate in severe sepsis. N Engl J Med.

[69] Zarychanski R, Abou-Setta AM, Turgeon AF (2013). Association of hydroxyethyl starch administration with mortality and acute kidney injury in critically Ill patients. A systematic review and meta-analysis. JAMA.

[70] Boyd JH, Frcp C, Forbes J (2011). Fluid resuscitation in septic shock: a positive fluid balance and elevated central venous pressure are associated with increased mortality. Crit Care Med.

[71] Abulebda K, Cvijanovich NZ, Thomas NJ (2014). Post-ICU admission fluid balance and pediatric septic shock outcomes: a risk-stratified analysis. Crit Care Med.

[72] Wong HR, Salisbury S, Xiao Q (2012). The pediatric sepsis biomarker risk model. Crit Care.

[73] Daniel De Backer M, Biston P, Devriendt J (2010). Comparison of dopamine and norepinephrine in the treatment of shock. N Engl J Med.

[74] De Backer D, Aldecoa C, Njimi H (2012). Dopamine versus norepinephrine in the treatment of septic shock: a meta-analysis*. Crit Care Med.

[75] Ventura AMC, Shieh HH, Bousso A (2015). Double-blind prospective randomized controlled trial of dopamine versus epinephrine as first-line vasoactive drugs in pediatric septic shock. Crit Care Med.

[76] Ramaswamy KN, Singhi S, Jayashree M (2016). Double-blind randomized clinical trial comparing dopamine and epinephrine in pediatric fluid-refractory hypotensive septic shock. Pediatr Crit Care Med.

[77] Deep A, Goonasekera CD, Wang Y (2013). Evolution of haemodynamics and outcome of fluid-refractory septic shock in children. Intensive Care Med.

[78] Belletti A, Musu M, Silvetti S (2015). Non-adrenergic vasopressors in patients with or at risk for vasodilatory shock. A systematic review and meta-analysis of randomized trials. PLoS One.

[79] Yildizdas D, Yapicioglu H, Celik U (2008). Terlipressin as a rescue therapy for catecholamine-resistant septic shock in children. Intensive Care Med.

[80] Choong K, Bohn D, Fraser DD (2009). Vasopressin in pediatric vasodilatory shock: a multicenter randomized controlled trial. Am J Respir Crit Care Med.

[81] Masarwa R, Paret G, Perlman A (2017). Role of vasopressin and terlipressin in refractory shock compared to conventional therapy in the neonatal and pediatric population: a systematic review, meta-analysis, and trial sequential analysis. Crit Care.

[82] Rios DR, Kaiser JR (2015). Vasopressin versus dopamine for treatment of hypotension in extremely low birth weight infants: a randomized, blinded pilot study. J Pediatr.

[83] Lee JH, Chan YH, Lai OF (2013). Vasopressin and copeptin levels in children with sepsis and septic shock. Intensive Care Med.

[84] Rutledge C, Brown B, Benner K (2015). A novel use of methylene blue in the pediatric ICU. Pediatrics.

[85] de Graaf H, Tebruegge M, Faust SN (2014). Evidence base for the use of corticosteroids in septic shock in children. Crit Care Med.

[86] Carcillo JA (2014). Evidence base for the use of corticosteroids in septic shock in children. Crit Care Med.

[87] Menon K, McNally D, Choong K (2013). A systematic review and meta-analysis on the effect of steroids in pediatric shock. Pediatr Crit Care Med.

[88] Menon K, McNally JD, Choong K (2013). A survey of stated physician practices and beliefs on the use of steroids in pediatric fluid and/or vasoactive infusion-dependent shock. Pediatr Crit Care Med.

[89] Menon K, Wong HR (2015). Corticosteroids in pediatric shock: a call to arms. Pediatr Crit Care Med.

[90] Annane D (2002). Effect of treatment with low doses of hydrocortisone and fludrocortisone on mortality in patients with septic shock. JAMA.

[91] Sprung C, Annane D, Keh D (2008). Hydrocortisone therapy for patients with septic shock. N Engl J Med.

[92] Annane D, Bellissant E, Bollaert P-E (2009). Corticosteroids in the treatment of severe sepsis and septic shock in adults. a systematic review. JAMA.

[93] Sligl WI, Milner DA, Sundar S (2009). Safety and efficacy of corticosteroids for the treatment of septic shock: a systematic review and meta-analysis. Clin Infect Dis.

[94] Patel GP, Balk RA (2012). Systemic steroids in severe sepsis and septic shock. Am J Respir Crit Care Med.

[95] Zimmerman JJ, Williams MD (2011). Adjunctive corticosteroid therapy in pediatric severe sepsis: observations from the RESOLVE study. Pediatr Crit Care Med.

[96] Maclaren G, Butt W, Best D (2007). Extracorporeal membrane oxygenation for refractory septic shock in children: one institution’s experience. Pediatr Crit Care Med.

[97] Maclaren G, Butt W, Best D (2011). Central extracorporeal membrane oxygenation for refractory pediatric septic shock. Crit Care Med.

[98] Skinner SC, Iocono JA, Ballard HO (2012). Improved survival in venovenous vs venoarterial extracorporeal membrane oxygenation for pediatric noncardiac sepsis patients: a study of the Extracorporeal Life Support Organization registry. J Pediatr Surg.

[99] Ruth A, McCracken CE, Fortenberry JD (2015). Extracorporeal therapies in pediatric severe sepsis: findings from the pediatric health-care information system. Crit Care.

[100] Smith S, Butt W, Best D (2016). Long-term survival after extracorporeal life support in children with neutropenic sepsis. Intensive Care Med.

[101] Foland JA, Fortenberry JD, Warshaw BL (2004). Fluid overload before continuous hemofiltration and survival in critically ill children: a retrospective analysis. Crit Care Med.

[102] Selewski DT, Cornell TT, Blatt NB (2012). Fluid overload and fluid removal in pediatric patients on extracorporeal membrane oxygenation requiring continuous renal replacement therapy. Crit Care Med.

[103] Modem V, Thompson M, Gollhofer D (2014). Timing of continuous renal replacement therapy and mortality in critically ill children*. Crit Care Med.

[104] de Galasso L, Emma F, Picca S (2016). Continuous renal replacement therapy in children: fluid overload does not always predict mortality. Pediatr Nephrol.

[105] Kawai Y, Cornell TT, Cooley EG (2015). Therapeutic plasma exchange may improve hemodynamics and organ failure among children with sepsis-induced multiple organ dysfunction syndrome receiving extracorporeal life support. Pediatr Crit Care Med.

[106] Rimmer E, Houston BL, Kumar A (2014). The efficacy and safety of plasma exchange in patients with sepsis and septic shock: a systematic review and meta-analysis. Crit Care.

[107] Rhodes A, Phillips G, Beale R (2015). The Surviving Sepsis Campaign bundles and outcome: results from the International Multicentre Prevalence Study on Sepsis (the IMPreSS study). Intensive Care Med.

[108] Han YY, Carcillo JA, Dragotta MA (2003). Early reversal of pediatric-neonatal septic shock by community physicians is associated with improved outcome. Pediatrics.

[109] Oliveira C, Nogueirade Sá F, Oliveira D (2008). Time- and fluid-sensitive resuscitation for hemodynamic support of children in septic shock. Barriers to the implementation of the American college of critical care medicine/pediatric advanced life support guidelines in a pediatric intensive care unit i. Pediatr Emerg Care.

[110] Inwald DP, Tasker RC, Peters MJ (2009). Emergency management of children with severe sepsis in the United Kingdom: the results of the Paediatric Intensive Care Society sepsis audit. Arch Dis Child.

[111] Paul R, Neuman MI, Monuteaux MC (2012). Adherence to PALS sepsis guidelines and hospital length of stay. Pediatrics.

[112] Paul R, Melendez E, Stack A (2014). Improving adherence to PALS septic shock guidelines. Pediatrics.

[113] Long E, Babl FE, Angley E (2016). A prospective quality improvement study in the emergency department targeting paediatric sepsis. Arch Dis Child.

[114] Akcan Arikan A, Williams EA, Graf JM (2015). Resuscitation bundle in pediatric shock decreases acute kidney injury and improves outcomes. J Pediatr.

[115] Balamuth F, Weiss SL, Fitzgerald JC (2016). Protocolized treatment is associated with decreased organ dysfunction in pediatric severe sepsis. Pediatr Crit Care Med.

[116] Nadel S, Goldstein B, Williams MD (2007). Drotrecogin alfa (activated) in children with severe sepsis: a multicentre phase III randomised controlled trial. Lancet.

[117] Farris RWD, Weiss NS, Zimmerman JJ (2013). Functional outcomes in pediatric severe sepsis: further analysis of the researching severe sepsis and organ dysfunction in children: a global perspective trial. Pediatr Crit Care Med.

[118] Aspesberro F, Mangione-Smith R, Zimmerman JJ (2015). Health-related quality of life following pediatric critical illness. Intensive Care Med.

[119] Lin JC, Spinella PC, Fitzgerald JC (2017). New or progressive multiple organ dysfunction sydrome in pediatric severe sepsis: a sepsis phenotype with higher morbidity and mortality. Pediatr Crit Care Med.

[120] Hartman M, Lin JC (2013). Functional outcomes for children with severe sepsis: is a “good save” good enough?. Pediatr Crit Care Med.

